# 10-Year Weight Gain in 13,802 US Adults: The Role of Age, Sex, and Race

**DOI:** 10.1155/2022/7652408

**Published:** 2022-05-06

**Authors:** Larry A. Tucker, Kayla Parker

**Affiliations:** College of Life Sciences, Brigham Young University, Provo, Utah 84602, USA

## Abstract

**Purpose:**

The primary objective of the present investigation was to identify 10-year weight gain patterns in 13,802 US adults and also to determine the extent that differences in 10-year weight gains were associated with the key demographic variables: age, sex, and race.

**Methods:**

The study design was cross-sectional and included the years 2011–2018. Data for 2019–2020 were not available because of COVID. A multistage random sampling strategy was employed. Specifically, individual sample weights and randomly selected clusters and strata were used with each statistical model, allowing the results to be generalized to the US adult population.

**Results:**

Mean (±SE) 10-year weight gain was 4.2 ± 0.2 kg or 6.6 ± 0.2% of initial body weight. A total of 51% of the participants gained 5% or more body weight, 36% gained 10% or more, and 16% gained 20% or more across the 10-years. Age was linearly and inversely associated with 10-year weight gain, expressed in kg (*F* = 166.4, *P* < 0.0001) or percent weight gain (*F* = 246.9, *P* < 0.0001), after adjusting for sex and race. For each 1-year increase in age, 10-year weight gain decreased by 0.20 ± 0.02 kg and 0.28 ± 0.02 percent. After adjusting for age and race, 10-year weight gain (kg) was significantly greater (*F* = 73.6, *P* < 0.0001) in women (5.4 ± 0.3) than in men (2.6 ± 0.2). Weight gain also differed across races, kg (*F* = 27.7, *P* < 0.0001) and % (*F* = 28.5, *P* < 0.0001). Non-Hispanic Blacks gained more weight and NH Asians gained less weight than the other races.

**Conclusion:**

Without question, 10-year weight gain is a serious problem within the US adult population. Younger adults, women, and Non-Hispanic Blacks, particularly Black women, seem to experience the highest levels of 10-year weight gain. Consequently, obesity and weight gain prevention programs focusing on these at-risk individuals should be a public health priority.

## 1. Introduction

The prevalence of obesity continues to increase in the United States. In 2017–2018, more than 42% of the adult population had obesity and almost 10% had severe obesity [1]. Unfortunately, the health consequences of obesity are serious and substantial. As seen within the clinical setting, obesity can be a deadly disease. Common outcomes associated with obesity include coronary heart disease, stroke, insulin resistance, dyslipidemia, osteoarthritis, hypertension, type 2 diabetes, and premature mortality, to name a few [2].

Obesity results from weight gain over time. Sadly, the prevalence of obesity continues to grow. Likewise, the obesity literature continues to expand as well. Although weight gain drives obesity, weight gain is studied less frequently than obesity. This is partly because obesity is an outcome that can be measured at a given point in time, whereas long-term weight gain is a process that must be assessed over time.

Like obesity, weight gain is linked with several serious diseases and increased mortality. For example, in a classic study of more than 50,000 men, the risk of type 2 diabetes was directly associated with absolute weight gain throughout adulthood [3]. In a study by Bassett et al., men were found to have a 13% greater risk of prostate cancer mortality for each 5 kg gain in body weight [4]. In a Norwegian cohort study with 19 years of follow-up, adults who gained 15 kg or more had 1.5 times the risk of CVD mortality than their counterparts, and adults whose weight gain moved them from normal weight to obese had more than twice the risk of CVD mortality compared to those who remained in the normal weight category [5]. In the Framingham Offspring Study, women and men who gained weight and progressed from normal weight to overweight during midlife had a 2.2 and 1.6-fold increased cancer risk, respectively, compared to those who remained at normal weight [6]. Lastly, in a recent study of almost 16,000 adults, a 10 kg increase in body weight was associated with significant and meaningful increases in systolic and diastolic blood pressures in men and women [7].

National data summarizing obesity trends in the USA are common [1, 8–11]. On the other hand, population-based findings of weight gain are rare, especially long-term weight gain. Hence, the purpose of the present study was to identify 10-year weight gain patterns representative of the US adult population. An additional objective was to determine the extent that 10-year weight gain differs among adults based on age, sex, and race. By determining weight gain trends and the extent that age, sex, and race predict long-term weight gain, clinicians and other health care providers can better identify at-risk individuals and reduce the risk of obesity over time.

## 2. Methods

### 2.1. Sample

The National Health and Nutrition Examination Survey (NHANES) is an ongoing research program administered by the National Center for Health Statistics, USA. The primary aim of NHANES is to assess the health and nutrition status of children and adults in the United States and to identify national trends over time using questionnaires, examinations, laboratory tests, and interviews. NHANES selects participants randomly using a four-stage, probability sampling design [12]. Outcomes are therefore generalizable to the US civilian, noninstitutionalized population, when individual sample weights, strata, and clusters are employed correctly within analyses. Although NHANES data have been collected for decades, the present investigation focused on the most recent 8 years of available data ending in 2018. Data for 2019–2020 are not available because of issues due to COVID.

Every participant was required to give written consent to participate in NHANES. Consent to collect individual data and post it online without identifying information was approved by the Ethics Review Board of the National Center for Health Statistics [13]. NHANES data are free and can be accessed by researchers and the general public online [14].

The sample was delimited to adults 36–79 years of age for two reasons. First, the focus of the study was 10-year weight change. If the typical NHANES definition of “adult” were employed (i.e., 20 years of age or older), then weight gain values beginning at 10 years of age would be necessary. If 30-year-old individuals were included, then weight gain calculations beginning at 20 years of age would be needed. This would be problematic because some 20-year-old individuals are still growing and developing physically. Consequently, a lower-end age limit of 36 years was used, consistent with other researchers who have studied 10-year weight change using NHANES data [15]. This allowed the 10-year weight change calculation to begin at age 26, so physical maturation was not an issue. Pregnant women were excluded.

### 2.2. Measurement Methods

In the present study, current body weight was measured and weight 10 years earlier was reported by participants. There were 3 exposure measures, each a key demographic variable: age, sex, and race. The outcome variable was weight change over the previous 10 years. Weight change was indexed using two definitions: weight change in kg as well as weight change expressed as a percentage of initial body weight.

#### 2.2.1. Age, Sex, and Race

Age in years was self-reported at the time of the screening. NHANES reported sex as male or female. Race/Hispanic origin was defined by NHANES as Mexican American, Other Hispanic, Non-Hispanic White, Non-Hispanic Black, Non-Hispanic Asian, or Other Race/Multiracial.

#### 2.2.2. 10-Year Weight Gain

Participants were weighed in kg using a digital scale [16]. The scale was calibrated each day using known weights. Participants wore a standard examination gown, consisting of a disposable shirt, pants, and slippers. Underpants were worn beneath the paper gown. If the individual weighed more than 200 kg (440 lbs), a second digital scale was used. The individual stood balanced with one foot on each scale and the combined weights were recorded [16]. To determine 10-year weight change, participants reported their body weight from 10 years earlier. Initial body weight was subtracted from their current weight to determine weight change (kg). Percent weight change was also calculated. Cut points of 5%, 10%, and 20% or more weight gain over the previous 10 years were used to describe the findings.

There is abundant evidence indicating that self-reported body weight closely aligns with measured weight. Specifically, an investigation of 1302 adults, published in the American Journal of Clinical Nutrition by Stunkard et al. [17], showed that self-reported body weight and measured weight in US adults are almost perfectly correlated (*r* = 0.99, *P* < 0.01). Stunkard concluded [17], “Self-reported weights were remarkably accurate across all these variables in the American sample, even among obese people, and may obviate the need for measured weights in epidemiological investigations” (p. 1593). Research by Perry et al. [18] and also two samples studied by Wing et al. resulted in correlations of 0.98 between self-reported and measured body weight [19].

Research based on looking back in time also shows very strong correlations between recalled weight and measured body weight. Using a sample of 1,805 US men, Rhoads et al. had participants recall their draft registration weights at age 25 when they were 20–30 years older [20]. On average, the recalled weights and draft registration weights differed by only 2.2%. Additionally, in a sample of 118 women, participants were asked to recall their weight at age 18 when each woman was between 25 and 42 years old, a gap in time of 7–24 years. Measured weights at age 18 were based on physical examinations. The weight difference was 1.4 kg and the correlation between the two was 0.87 (*P* < 0.01).

In a Harvard study, recalled and measured height and weight measurements were utilized to compare BMI differences in 181 men and women [21]. Adults aged 71–76 were asked to recall their heights and weights in their last year of high school. The authors concluded, “Means and standard deviations of recalled and measured values of weight and height were in close agreement” (p. 59) [21]. According to Willett [22], the correlation between recalled values and measured BMIs approximately 55 years later was 0.91 (*P* < 0.01) for men and 0.92 (*P* < 0.01) for women. Finally, in his textbook titled Nutritional Epidemiology [22], Willett states that “recalled weight from many years earlier appears to be highly valid,” (p. 247), and differences between recalled and measured values “have minimal effect on epidemiologic measures of association” (p. 247).

A few years ago, Veldheer et al. published a paper in the International Journal of Obesity using the same NHANES, 10-year weight change variable employed in the present study [15]. Consistent with the literature on smoking and weight change, Veldheer's investigation showed that never smokers, current smokers, and ex-smokers differed significantly in weight change over the previous 10 years [15].

### 2.3. Statistical Analysis

NHANES assigned each participant an individual sample weight based on census data, and each weight indicated the number of people in the United States characterized by the sample person. The sample weights represented the “differential probabilities of selection for individual domains, nonresponse to survey instruments, and differences between the final sample and the total population” (p. 7) [23]. Impartial U.S. estimates were produced because each statistical analysis was conducted using the sample weights. Findings from the present investigation can be generalized to the noninstitutionalized, civilian adult population in the United States because clusters, strata, and individual sample weights were used in the analyses.

For categorical variables, frequencies were calculated to describe the data, and for continuous variables, means (±SE) were employed. To measure the degree that gains in weight over the previous 10 years differed across categories of age, sex, and race, analysis of variance (ANOVA) was performed using regression analysis. Partial correlation and the SAS SurveyReg procedure were used to control for differences in sex and race when age was studied as the exposure variable. Likewise, age and race were controlled when sex was employed as the exposure variable, and adjustments were made for age and sex when differences in long-term weight gain across categories of the race were studied. Variance estimations were generated using the Taylor method.

Some researchers assume that statistical power is extremely high when using the large samples that are commonly associated with NHANES data sets. This is not true. Because NHANES uses a multistage random sampling procedure, which allows the results to be generalized broadly to US adults, each analysis in the present study was performed with 62 degrees of freedom in the denominator. The 62 degrees of freedom were derived by subtracting the 59 strata from the 121 clusters. Statistical significance was acknowledged when alpha was less than 0.05, and all *P* values were two-sided. SAS version 9.4 (SAS Institute, Inc., Cary, NC) was employed to perform the statistical tests.

## 3. Results

There were 13,802 participants in the study. Women comprised 52.0% of the sample. The average (±SE) age was 54.6 ± 0.2 years and the mean 10-year weight gain was 4.2 ± 0.2 kg or 6.6 ± 0.2% of initial body weight. Prevalence and weighted percentages for the categorical variables are shown in Table 1.

As shown in Table 1, more than 50% of the sample gained 5% or more in body weight over the previous 10 years. Over 35% gained 10% or more, and 16% gained 20% or more across the previous 10 years. Mean body weight initially was 80.1 ± 0.3 kg, and 10 years later, the mean weight was 84.3 ± 0.3 kg. Initially, the median weight was 77.0 ± 0.5 kg and 10 years later it was 81.5 ± 0.3 kg. Mean BMI was 28.2 ± 0.1 initially and 29.7 ± 0.1 10 years later.  Table 2 displays the weighted percentiles (±SE), including the 5^th^, 25^th^, 50^th^, 75^th^, and 95^th^ percentiles, for the continuous variables.

When treated as a continuous variable, age was linearly and inversely associated with weight gain, expressed in kg (*F* = 166.4, *P* < 0.0001) or percent weight gain (*F* = 246.9, *P* < 0.0001), after adjusting for sex and race within the total sample. Specifically, for each 1-year increase in age, 10-year weight gain decreased by 0.20 ± 0.02 kg and 0.28 ± 0.02 percent, on average. Focusing only on women, the relationships between age and 10-year weight gain (kg) and (%), respectively, were virtually identical to the associations within the combined sample (*F* = 97.9, *P* < 0.0001; regression coefficient: −0.20 ± 0.02 kg) and (*F* = 147.8, *P* < 0.0001; regression coefficient: −0.30 ± 0.02 percent). Within men only, the relationships between age and 10-year weight gain (kg) and (%), respectively, were similar to the findings within the total sample (*F* = 111.1, *P* < 0.0001; regression coefficient: −0.21 ± 0.02 kg) and (*F* = 152.2, *P* < 0.0001; regression coefficient: −0.25 ± 0.02 percent).

With sex and race controlled, and age treated as a categorical variable, mean weight gain (kg) and mean percent weight gain (%) differed significantly across the age categories, within women only, men only, and within the combined sample.  Table 3 shows the mean differences in 10-year weight gain across the age categories.

As displayed in Table 3, within the total sample, each 10-year weight gain mean, expressed in kg or as a percentage, differed from each other mean in a dose-response manner. Specifically, adults in the 36–39 age category gained the most weight during the previous 10 years, followed by those 40–49 years old, followed by those 50–59 years old, etc. Adults in the 70–79 age category gained the least amount of weight during the previous 10 years. Weight gain differences were more pronounced when expressed as percent weight gained compared to absolute weight gained (kg).

The dose-response relationship between age and weight gain was clear in the total sample, and it held true in the men-only analyses. However, in the women-only comparisons, there was no significant difference in mean 10-year weight gain (kg or %) between the first two age categories, 36–39 and 40–49, as shown in Table 3.

According to Table 4, after adjusting for differences in age and race, 10-year weight gain was significantly greater in women than men. The 10-year weight gain difference was greater when expressed as percent weight gain (%) compared to absolute weight gain (kg). US women gained about twice the absolute weight (kg) as men over the previous 10 years and 2.4 times more weight when displayed as percent weight gain.

As shown in Table 5, 10-year weight gain differed significantly across the NHANES race categories. Specifically, within the total sample, Non-Hispanic Blacks were found to have significantly greater weight gain, kg and %, than any other race, except the Other or Multirace group. This group was small, representing only about 3% of the US population, and included substantial variation, as noted by the larger standard error values.

Although Non-Hispanic (NH) Blacks consistently had the largest weight gain during the previous 10 years, NH Asians consistently had the smallest weight gain compared to the other race categories. Within the total sample, NH Blacks experienced more than 3 times the 10-year weight gain compared to NH Asians, and more than 50% greater weight gain than any other NHANES racial group. Mexican Americans, Other Hispanics, NH Whites, and Other/Multiracial individuals did not differ in long-term weight gain.

When separated based on sex, 10-year weight gain differences were more significant across racial groups in women (*F* = 28.5, *P* < 0.0001) than men (*F* = 4.6, *P* < 0.0001). Specifically, NH Black women experienced more than triple the weight gain (kg) compared to NH Asians, 73% more than NH Whites, and 50% more than Mexican American women. On the other hand, NH Black men did not gain more weight than Mexican American, Other Hispanic, or Other/Multiracial men. Weight gain differences between NH Black men and NH White or NH Asian men were similar to differences in women, as shown in Table 5.

## 4. Discussion

Evaluation of national US 10-year weight gain trends was one of the purposes of the present investigation. A second objective was to determine the degree that 10-year weight gain in the USA differs among adults based on age, sex, and race, with the expectation that health professionals will more accurately identify at-risk individuals and prevent weight gain to decrease the widespread plague of obesity.

Several important findings stand out in the present study. First, US adults tend to gain significant amounts of weight over the preceding 10 years. Mean 10-year weight gain was 4.2 ± 0.2 kg or 6.6 ± 0.2% of initial body weight within the United States. Additionally, more than 50% of US adults gained 5% or more in body weight over the previous 10 years. Over 35% gained 10% or more, and 16% gained 20% or more across the previous 10 years. Second, 10-year weight gain is strongly related to age. The association is linear and inverse. The greatest gains in weight are in young and middle-aged adults, and less weight is gained as age increases. Third, 10-year weight gain is substantially greater in US women compared to men. On average (±SE), women gained 5.4 ± 0.3 kg and 9.2 ± 0.4 percent of their initial weight over the previous 10 years, whereas men gained 2.6 ± 0.2 kg and 3.8 ± 0.3 percent of their initial weight. In general, compared to US men, women gained about twice as much weight (kg) and 2.4 times more weight expressed as a percent of initial weight, over the previous 10 years. Fourth, 10-year weight gain is significantly higher in Non-Hispanic Blacks than in other racial groups, especially NH Black women. Moreover, 10-year weight gain is significantly lower in Non-Hispanic Asians compared to other racial categories.

Current national data, generalizable to the civilian, noninstitutionalized adult population, indicates that 10-year weight gain is a serious problem in the United States. As a consequence of the large amounts of weight gained in the adult years, the prevalence of obesity in the USA is dangerously elevated. The most recent findings from the U.S. Department of Health and Human Services and the Centers for Disease Control and Prevention (2017–2018) show that 42.4% of US adults have obesity and almost 10% have severe obesity [1]. In 1999-2000, the prevalence of obesity was 30.5% and severe obesity was 4.7%. In less than 20 years, the prevalence of obesity increased by approximately 40%, and severe obesity almost doubled [1].

The national 10-year weight gain results of the present study show an inverse, dose-response relationship with age. In short, 10-year weight gain is highest in young adults and weight gain tends to decrease with each decade of age. This seems to conflict with national obesity findings [1]. National results indicate that the prevalence of obesity tends to increase with age until adults reach the 60-and-over age category. Then, the prevalence of obesity tends to decline slightly, probably because the higher mortality rate of older individuals with obesity reduces the number of adults with obesity.

Why would 10-year weight gain be inversely related to age, whereas obesity is directly related to age? There are multiple reasons. First, it takes time for body weight to accumulate. On average, US adults gain roughly 8 kg from their late 20s to their late 30s. For most adults, this is not sufficient to cause obesity. However, another 6.5 kg is typically gained during the next decade. For some, this additional weight gain is enough to move the individual into the obese category. If not, adults tend to gain another 4.3 kg from their 40s to their 50s, moving a larger number of adults into the obese category. If not, another 2.1 kg is gained from the 50s to the 60s, on average. Overall, if an adult gains the average amount of weight during each decade of adult life, the total equals a substantial amount of weight.

Another reason weight gain is inversely related to age while obesity rates are directly related to age is that metabolism increases with weight gain. Larger adults have higher metabolisms than smaller adults. Just as it takes more fuel to maintain a larger house than a smaller one, individuals with obesity require more fuel to maintain their weight than nonobese individuals. Consequently, as body weight increases, it takes an increasing amount of energy (calories) to cause more weight gain. As a result, weight gain tends to slow with each decade of age in the average adult.

In a 2020 study by Jacobsen et al. [24], two population-based investigations were joined, the first, SAMINOR 1 (2003–2004), and the second, SAMINOR 2 (2012–2014). A total of 3496 adults participated in both surveys. All were Norwegian and 41% were Sami. Similar to the present study, in both men and women, 10-year weight gain was the highest in the youngest age group (ages 36–39) and progressively decreased with age (*P* < 0.0001). Similar findings were revealed in a study by Tanamas et al. that tracked weight gain over a 12-year period in Australian men and women [25]. From age 25–34 to over 75 years, weight gain decreased in a dose-response manner with increasing age (*P* < 0.001), like in the present investigation.

As shown in Tables 3 and 4, mean 10-year weight gain is significantly greater in US women than men across every decade of adult life, on average. The difference is greater when weight gain is defined using percent of initial weight rather than an absolute increase in kg, mainly because women tend to be smaller and lighter than men, so an equal weight gain (kg) results in a greater weight gain percentage. From the 6^th^ to the 7^th^ decade of life, national data indicate that US men actually lose body weight and women experience less weight gain compared to other decades. Loss of muscle mass and increasing frailty are likely contributing factors to these changes [26, 27].

In the study by Jacobsen et al. referred to earlier [24], two investigations (SAMINOR 1 AND 2) were paired together, and 10-year weight gains were determined for 3496 adults. Across the 10-year period, the Norwegian men experienced more weight gain than the women. These findings are inconsistent with the present US results, which show that women gain more weight than men over time. In an Australia-based study covering weight gain over a dozen years, there was no difference in weight gain between women and men [25].

The difference in weight gain between US women and men is often accounted for by the weight gained during pregnancy in women. In a 2019 study by Zoet et al., of 2,459 women followed prospectively, 1.5–2.0 kg of excess weight was gained per child, on average [28]. In a 2014 longitudinal study by Arabshahi et al., parity was associated significantly with weight gain [29]. However, the relationship was not linear. Research by the National Research Council and the Institute of Medicine indicates that each successive birth increases postpartum weight by 1 kg above that which is normally gained with age. Not all studies support this theory, however [30].

Weight gain results from an imbalance between energy consumption and energy expenditure. An important component of energy expenditure is physical activity. Research consistently shows that physical activity helps to prevent weight gain [31]. Multiple studies indicate that women tend to be less physically active than men [32–34]. Using NHANES data collected by accelerometers, Hawkins et al. found that objectively measured physical activity is substantially higher in US men than women [33], which could account for some of the 10-year weight gain difference between women and men.

Based on the national findings of the present study, race is a good predictor of 10-year weight gain. NH Blacks consistently experience the greatest 10-year weight gain and NH Asians show the smallest gain compared to other race categories. In general, differences in 10-year weight gain across other racial groups are similar. When studied separately, weight gain differences among the NHANES racial groups are more pronounced in women than men.

Weight gain drives obesity, and in the United States, prevalence rates of obesity continue to move further away from the Healthy People 2020 goal of 30.5% [1]. For US women, national obesity rates are consistent with the US 10-year weight gain findings of the present study. According to the latest national data (2017–2018), 56.9% of NH Black women have obesity, whereas 43.7% of Hispanic, 39.8% of NH White, and only 17.2% of NH Asian women have obesity [1]. Although the racial categories are labeled a little differently, the rank order of the racial groups for weight gain in US women is the same as for the prevalence of obesity. Moreover, NH Blacks and NH Asian women represent the extremes for both rates of 10-year weight gain and obesity [1].

For US men, rates of 10-year weight gain and obesity are not as aligned. Hispanics (45.7%) and NH Whites (44.7%) have higher rates of obesity than NH Black men (41.1%) [1], but NH Black men have the highest 10-year weight gains, according to the present investigation. Consistent with the weight gain findings, NH Asian men have substantially lower rates of obesity (17.5%) compared to other racial groups.

There are several factors that could explain, in part, why race is a good predictor of 10-year weight gain. First, there are significant social economic (SES) disparities linked to obesity [35]. In a meta-analysis of 24 prospective cohort studies, Kim et al. showed that less educated adults have a 33% higher risk of having overweight or obesity than their counterparts [36]. In an older US study, Kahn et al. found that women with very low family incomes have 1.7 times greater odds of major weight gain compared to their counterparts [37]. Men were not part of Kahn's study.

Lifestyle could be another reason that race is associated with 10-year weight gain. Specifically, physical activity differences could be a significant factor [38, 39]. The literature shows that regularly active adults are less likely to gain weight than their counterparts [31]. Using accelerometers to objectively measure physical activity, national findings indicate that approximately 59% of NH Blacks do not perform any moderate or vigorous physical activity during a week of monitoring, whereas 52% of NH Whites and 49% of Mexican Americans are totally inactive [32]. Kwon et al. found an inverse association between self-reported moderate to vigorous physical activity and obesity in NH White men and women and Black and Hispanic women, but no relationship in NH Asians [40].

Another lifestyle factor could be diet. Research shows that the types of food consumed differ across racial groups and may influence energy intake [41]. NH Black children are more likely to consume sugary drinks at home than others [42]. However, the degree to which this habit carries over to adulthood is not known. Additionally, higher-income adults are more likely to consume low-energy-dense foods, such as fruits and vegetables, and are also more likely to meet dietary guidelines for whole-grain intake than their counterparts [43, 44].

Finally, food insecurity has been proposed as a reason for racial disparities in obesity. A majority of investigations suggest there is a positive relationship between food insecurity and obesity [45], especially in minority women. Using NHANES data, Hernandez et al. showed that food insecurity is most prevalent in NH Blacks and Hispanics, regardless of sex. The investigation indicated that women with food insecurity are more likely to have overweight or obesity. However, food insecurity does not appear to predict overweight or obesity in men.

The current investigation had multiple limitations. First, because a randomized controlled trial could not be employed, conclusions implying causation are not appropriate. However, because age, sex, and race cannot be manipulated, randomized controlled trials or quasi-experimental investigations cannot be utilized to answer the research questions addressed in this study. Additionally, some of the outcomes of the present study could be a function of residual confounding. In other words, because age, sex, and race are related to a variety of variables predictive of weight gain, the associations uncovered in the present study could be partially a result of other factors, in addition to age, sex, and race.

There were multiple strengths and unique characteristics associated with this study. First, subjects were selected randomly from within the United States using a sophisticated multistage sampling strategy. Therefore, findings are generalizable to the civilian, noninstitutionalized US adult population. Second, the random sample was large and included 13,802 adults. Third, 10-year weight gain was not assessed in young adults, so outcomes were not affected by physical growth or maturation. Fourth, other investigations using samples representing US adults and 10-year weight gains as they relate to age, sex, and race are extremely rare in the literature.

## 5. Conclusion

In conclusion, findings from the present study, based on a nationally representative sample of 13,802 adults, indicate that 10-year weight gain in US women and men is substantial. Age, sex, and race each account for differences in 10-year weight gain. The relationship between age and weight gain is inverse and dose-response. Younger adults gain more weight than middle-aged adults, and middle-aged adults gain more than older adults. Moreover, US women gain more weight than men during each decade of adult life. Lastly, 10-year weight gain is the highest in Non-Hispanic Blacks and lowest in Non-Hispanic Asians. Although long-term weight gain appears to be a problem with an alarming number of US adults, younger adults, women, and Non-Hispanic Blacks, particularly Black women, clearly experience the most weight gain. By determining weight gain patterns and the extent that age, sex, and race predict long-term weight gain, clinicians, practitioners, and educators can better identify at-risk individuals. Knowing who is more likely to gain weight can help health care providers and public health officials focus more on at-risk individuals and use appropriate resources to promote prevention strategies to curb the widespread epidemic of obesity.

## Figures and Tables

**Table 1:** Descriptive characteristics of the sample (*n* = 13,802).

Categorical variable	*N*	Weighted %	SE
Age (years)			
36–39	1347	10.3	0.4
40–49	3415	26.8	0.6
50–59	3425	28.3	0.6
60–69	3578	22.2	0.6
70–79	2037	12.4	0.4
Sex			
Men	7108	48.0	0.5
Women	6694	52.0	0.5
Race			
Mexican American	1847	7.3	0.8
Other Hispanic	1535	5.7	0.6
Non-Hispanic White	4881	67.6	1.7
Non-Hispanic Black	3274	10.8	0.9
Non-Hispanic Asian	1815	5.3	0.5
Other Race/Multiracial	450	3.3	0.3
Gained 5% or more			
No	6846	49.0	0.6
Yes	6956	51.0	0.6
Gained 10% or more			
No	8769	64.3	0.6
Yes	5033	35.7	0.6
Gained 20% or more			
No	11407	84.0	0.4
Yes	2396	16.0	0.4

*N* refers to the number of subjects within the category. SE refers to the standard error of the percentage. The variable “Gained 5% or more” indicates that participants gained 5% or more in body weight from the initial period to the body weight assessment taken 10 years later. “Gained 10%” and “Gained 20%” are similarly defined. The weighted % column shows the distribution of subjects after the NHANES sample weights were applied. The weighted % values are more meaningful than the number of subjects (*N*) because the weighted % values take into account the sample weights and therefore can be generalized to the US adult population.

**Table 2:** Percentile distributions of the key variables representing US women and men.

Variable	Percentile (±SE)				
	5th	25th	50th	75^th^	95th
Age (years)					
Women (*n* = 7108)	36.8 ± 0.1	44.6 ± 0.3	53.8 ± 0.4	63.1 ± 0.2	74.2 ± 0.2
Men (*n* = 6694)	36.9 ± 0.0	44.5 ± 0.4	53.6 ± 0.4	62.5 ± 0.4	73.6 ± 0.3
Combined (*n* = 13,802)	36.8 ± 0.0	44.6 ± 0.3	53.7 ± 0.3	62.8 ± 0.3	73.9 ± 0.2
10-year weight change (kg)					
Women (*n* = 7108)	−16.1 ± 1.1	−1.0 ± 0.3	5.2 ± 0.2	12.4 ± 0.3	28.8 ± 0.8
Men (*n* = 6694)	−16.5 ± 0.5	−3.6 ± 0.3	2.6 ± 0.2	9.0 ± 0.2	22.5 ± 0.6
Combined (*n* = 13,802)	−16.2 ± 0.6	−2.4 ± 0.2	4.0 ± 0.1	10.8 ± 0.2	26.2 ± 0.5
10-year weight change (%)					
Women (*n* = 7108)	−18.3 ± 0.9	−1.6 ± 0.4	7.7 ± 0.3	18.1 ± 0.4	41.7 ± 1.1
Men (*n* = 6694)	−16.5 ± 0.5	−4.3 ± 0.3	3.0 ± 0.2	10.7 ± 0.3	26.8 ± 0.8
Combined (*n* = 13,802)	−17.2 ± 0.5	−3.1 ± 0.2	5.3 ± 0.2	14.6 ± 0.2	35.7 ± 0.5
Initial body weight (kg)					
Women (*n* = 7108)	49.8 ± 0.5	58.9 ± 0.5	67.8 ± 0.5	81.4 ± 0.6	113.2 ± 1.1
Men (*n* = 6694)	63.3 ± 0.5	74.9 ± 0.5	84.8 ± 0.5	99.6 ± 0.6	120.3 ± 1.1
Combined (*n* = 13,802)	52.2 ± 0.4	64.6 ± 0.5	77.0 ± 0.5	90.7 ± 0.6	117.6 ± 1.1
10-year body weight (kg)					
Women (*n* = 7108)	51.1 ± 0.6	63.4 ± 0.4	74.3 ± 0.5	88.7 ± 0.5	119.2 ± 1.3
Men (*n* = 6694)	63.6 ± 0.5	77.3 ± 0.4	88.7 ± 0.5	101.8 ± 0.5	128.2 ± 1.2
Combined (*n* = 13,802)	54.8 ± 0.5	69.1 ± 0.3	81.5 ± 0.3	96.0 ± 0.5	123.8 ± 0.8

SE: standard error of the percentile. Table values include person-level weighted adjustments based on the sampling methods of NHANES, so values represent those of the US adult population.

**Table 3:** Mean differences in 10-year weight gain across age categories in US women and men, after adjusting for the covariates.

Outcome	Age categories					*F*	*P*
	36–39 years	40–49 years	50–59 years	60–69 years	70–79 years		
Women only (*n* = 7108)							
10-year weight gain (kg)	9.0^a^ ± 0.7	7.7^a^ ± 0.5	5.9^b^ ± 0.6	3.3^c^ ± 0.6	1.8^d^ ± 0.5	30.2	<0.0001
10-year weight gain (%)	14.5^a^ ± 0.9	12.8^a^ ± 0.7	10.1^b^ ± 0.8	6.5^c^ ± 0.8	3.7^d^ ± 0.6	46.4	<0.0001
Men only (*n* = 6694)							
10-year weight gain (kg)	6.5^a^ ± 0.7	5.0^b^ ± 0.4	2.7^c^ ± 0.4	0.9^d^ ± 0.5	−1.1^e^ ± 0.5	33.7	<0.0001
10-year weight gain (%)	8.5^a^ ± 0.7	6.8^b^ ± 0.5	3.9^c^ ± 0.4	1.7^d^ ± 0.5	−0.6^e^ ± 0.5	45.2	<0.0001
Combined (*n* = 13,802)							
10-year weight gain (kg)	7.8^a^ ± 0.5	6.4^b^ ± 0.3	4.3^c^ ± 0.4	2.1^d^ ± 0.4	0.3^e^ ± 0.4	52.6	<0.0001
10-year weight gain (%)	11.5^a^ ± 0.6	9.8^b^ ± 0.4	7.0^c^ ± 0.4	4.1^d^ ± 0.5	1.5^e^ ± 0.4	76.2	<0.0001

10-year weight gain is the average amount of weight gained over the previous 10 years, expressed in kg or as a percentage of initial body weight. Means on the same row with different superscript letters ^(a,b,c,d,e)^ are significantly different (*P* < 0.05). SE represents the standard error of the mean. Means have been adjusted for differences in race for the women only and men only analyses, and sex and race when the analysis used the entire sample. The mean difference between 10-year weight gain (kg) for those in the 36–39 and 40–49 age categories, using the combined sample, was significant at the *P*=0.0854 level. Across the five categories of age, the sample size percentages (%) and sample size numbers (n), when combined, were 36–39 years (10.3%, *n* = 1347), 40–49 years (26.8%, *n* = 3415), 50–59 years (28.3%, *n* = 3425), 60–69 years (22.2%, *n* = 3578), and 70–79 years (12.4%, *n* = 2037). Because NHANES sample weights were applied to each individual, differences in the sample size of each category should be interpreted based on the percentages (%), not the raw number (*n*). The sample size percentages (%) can be generalized to the US adult population, but the sample numbers (*n*) cannot.

**Table 4:** Mean differences in 10-year weight gain between women and men, after adjusting for the covariates.

Outcome	Mean ± SE		*F*	*P*
	Women	Men		
10-year weight gain (kg)	5.4 ± 0.3	2.6 ± 0.2	73.6	<0.0001
10-year weight gain (%)	9.2 ± 0.4	3.8 ± 0.3	194.1	<0.0001

10-year weight gain represents the average amount of weight gained over the previous 10 years, expressed in kg or as a percentage of initial body weight. SE = standard error of the mean. Means have been adjusted for differences in age and race. The sample size percentages (%) and number (*n*) were women (52.0%, *n* = 7108) and men (48.0%, *n* = 6694). Differences in the sample sizes for women and men should be interpreted based on the percentages (%), not the number (*n*), for the individual sample weights to have their effect.

**Table 5:** Differences in 10-year weight gain across U.S. races, separated by sex, after adjusting for the covtablecaptionariates.

Outcome	Mexican American Mean ± SE	Other Hispanic Mean ± SE	Non- Hispanic white Mean ± SE	Non- Hispanic black Mean ± SE	Non- Hispanic Asian Mean ± SE	Other or multi- racial Mean ± SE	*F*	*P*
Women only (*n* = 7108)								
10 yr weight gain (kg)	5.8^a^ ± 0.5	5.3^a^ ± 0.5	5.1^a^ ± 0.4	8.8^b^ ± 0.5	2.8^c^ ± 0.3	4.9^a^ ± 2.1	28.5	<0.0001
10 yr weight gain (%)	9.9^a^ ± 0.7	9.6^a^ ± 0.7	8.5^a^ ± 0.4	13.4^b^ ± 0.6	5.6^c^ ± 0.4	9.6^a^ ± 2.6	29.9	<0.0001
Men only (*n* = 6694)								
10 yr weight gain (kg)	2.5^a,b^ ± 0.5	2.6^a,b^ ± 0.7	2.3^b^ ± 0.2	3.6^a^ ± 0.4	1.3^c^ ± 0.3	2.6^a,b,c^ ± 1.1	4.6	<0.0013
10 yr weight gain (%)	3.8^a,b,c^ ± 0.6	4.6^a,b^ ± 0.8	3.2^a,c^ ± 0.3	4.9^b^ ± 0.4	2.6^c^ ± 0.4	3.1^a,b,c^ ± 1.2	3.8	<0.0049
Combined (*n* = 13802)								
10 yr weight gain (kg)	4.1^a^ ± 0.3	4.0^a^ ± 0.4	3.7^a^ ± 0.2	6.3^b^ ± 0.3	2.0^c^ ± 0.2	3.8^a,b,c^ ± 1.2	27.7	<0.0001
10 yr weight gain (%)	6.8^a^ ± 0.5	7.0^a^ ± 0.5	5.8^a^ ± 0.2	9.3^b^ ± 0.4	4.0^c^ ± 0.3	6.3^a,b,c^ ± 1.5	28.5	<0.0001

Note: means on the same row with the same superscript letter ^(a,b,c)^ were not statistically different (*P* > 0.05). SE = standard error of the mean. Means were adjusted for differences in age, and with the sample combined, age and sex. Alpha was 0.0675 for the mean difference between Mexican American and NH Asian men. When combined, the sample size percentages (%) and sample size numbers (*n*) were: Mexican American (7.3%, *n* = 1847), other Hispanic (5.7%, *n* = 1535), NH white (67.6%, *n* = 4881), NH black (10.8%, *n* = 3274), NH Asian (5.3%, n=1815), and other or multi-racial (3.3%, *n* = 450). Because NHANES sample weights were applied to the percentages, differences in the sample sizes across the race categories should be interpreted based on the percentages (%), not the number, (*n*).

## Data Availability

All data supporting reported results can be found online as part of the National Health and Nutrition Examination Survey (NHANES). The data are free and can be found at the following website: https://wwwn.cdc.gov/nchs/nhanes/Default.aspx.
